# Perforation of small intestinal metastasis of lung adenocarcinoma treated with pembrolizumab: a case report

**DOI:** 10.1186/s40792-019-0730-6

**Published:** 2019-10-30

**Authors:** Shoki Sato, Naoto Senmaru, Keita Ishido, Takahiro Saito, Saseem Poudel, Jun Muto, Yasuhito Syouji, Ryunosuke Hase, Satoshi Hirano

**Affiliations:** 1 Department of Surgery, Steel Memorial Muroran Hospital, 1-45, Chiribetu-chou, Muroran, Hokkaido 050-0046 Japan; 2 Department of Gastroenterological Surgery II, Hokkaido University Faculty of Medicine, N-15 W-7, Sapporo, Hokkaido 060-8638 Japan

**Keywords:** Pembrolizumab, Small intestinal perforation, Non-small cell lung carcinoma metastasis

## Abstract

**Background:**

Pembrolizumab is an immune checkpoint inhibitor and is an anti-human programmed cell death-1 (PD-1) monoclonal antibody. Pembrolizumab is used for non-small cell lung carcinoma with high programmed cell death ligand-1 (PD-L1) expression. It has been found that better overall survival can be obtained using pembrolizumab compared to the existing chemotherapy. We report a case of perforation of small intestinal metastasis after pembrolizumab treatment.

**Case presentation:**

A 62-year-old man was treated by pembrolizumab for PD-L1 highly expressed lung adenocarcinoma, with multiple metastasis (small intestinal, lymph nodes, and bone). The treatment was stopped owing to drug-induced pneumonitis. One month after drug withdrawal, the patient visited the emergency department of our hospital with the complaint of severe stomachache. He had a rigid abdomen and generalized tenderness, and computed tomography scans showed free air within the abdomen. We diagnosed bowel perforation and performed emergency surgery. Surgical findings revealed multiple small intestine metastasis and mesenteric lymph node metastasis. Perforation was found in the metastatic site in the jejunum located around 40 cm anal to Treitz’s ligament. This perforated part was resected, and functional end-to-end anastomosis was performed using linear staplers. The post-operative course was uneventful. Pathological examination revealed lung adenocarcinoma metastasis at the perforation site, and the effectiveness of pembrolizumab was grade 1b (*Japanese Classification of the Colorectal Carcinoma*, seventh edition).

**Conclusions:**

This is the first report of perforation of small intestinal metastasis of lung adenocarcinoma after pembrolizumab treatment. Because pembrolizumab causes some side effects, particularly autoimmune side effects, careful attention during treatment is warranted.

## Background

Pembrolizumab, an immune checkpoint inhibitor, is an anti-human programmed cell death-1 (PD-1) monoclonal antibody and used for the treatment of non-small cell lung carcinoma (NSCLC) and melanoma with high expression of programmed cell death ligand-1 (PD-L1) and high microsatellite instability (MSI) status solid cancer [1–3].

Anti-PD-1 antibodies, including pembrolizumab, are reported to have not only general chemotherapy side effect, for example nausea, leukopenia, and more, but also characteristic autoimmune side effects like hypothyroidism, type 1 diabetes, hypopituitarism, colitis, and drug-induced pneumonitis due to excessive immune reaction [4, 5]. However, intestinal perforation caused by this drug has rarely been reported. We report a case of perforation of small intestinal metastasis of lung adenocarcinoma after pembrolizumab treatment.

## Case presentation

**Fig. 1:**
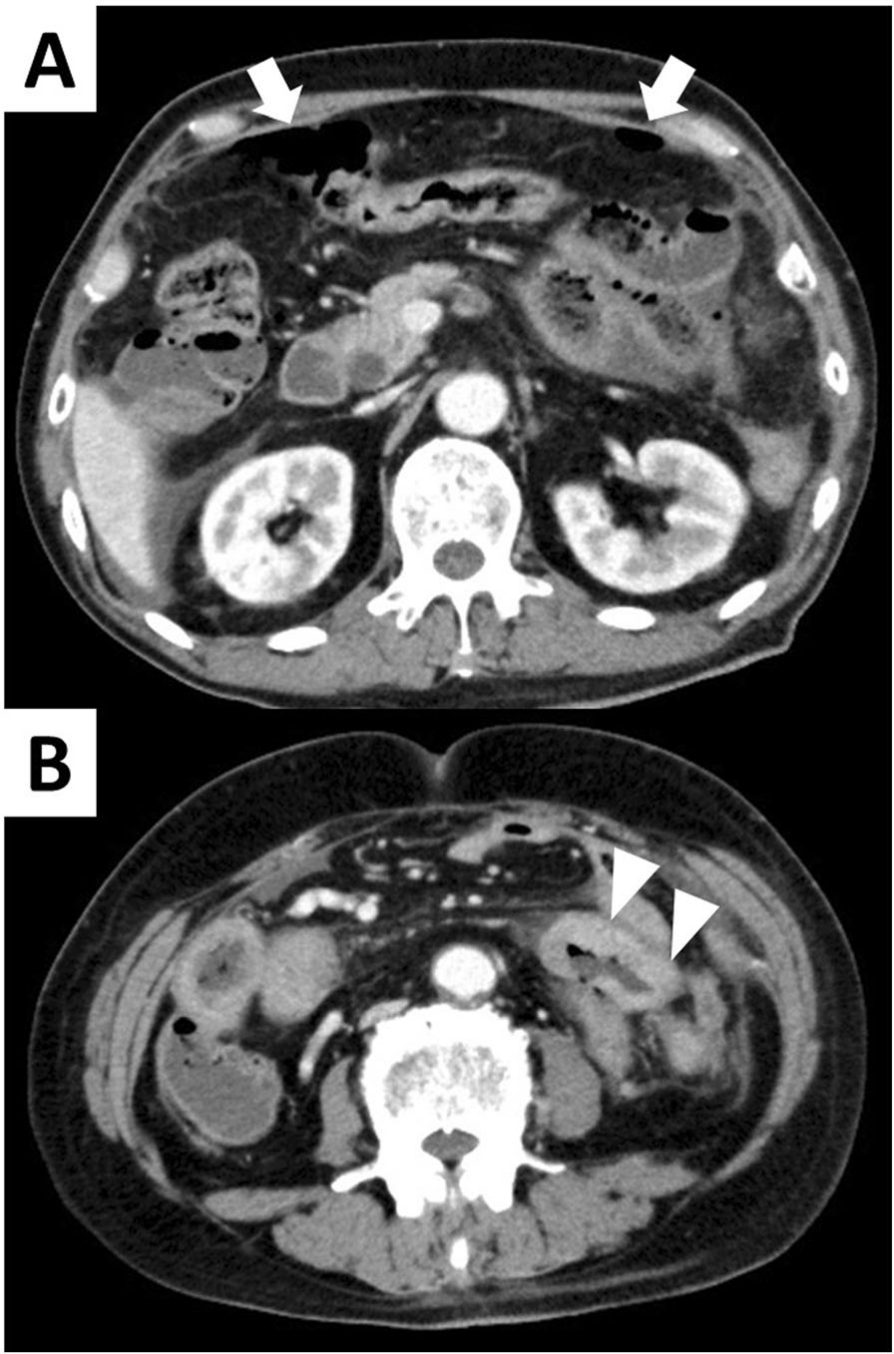
**a**, **b** Abdominal enhanced CT pictures are shown. Arrows show abdominal free air, and triangles show the perforation site of small intestinal metastasis of lung adenocarcinoma

**Fig. 2:**
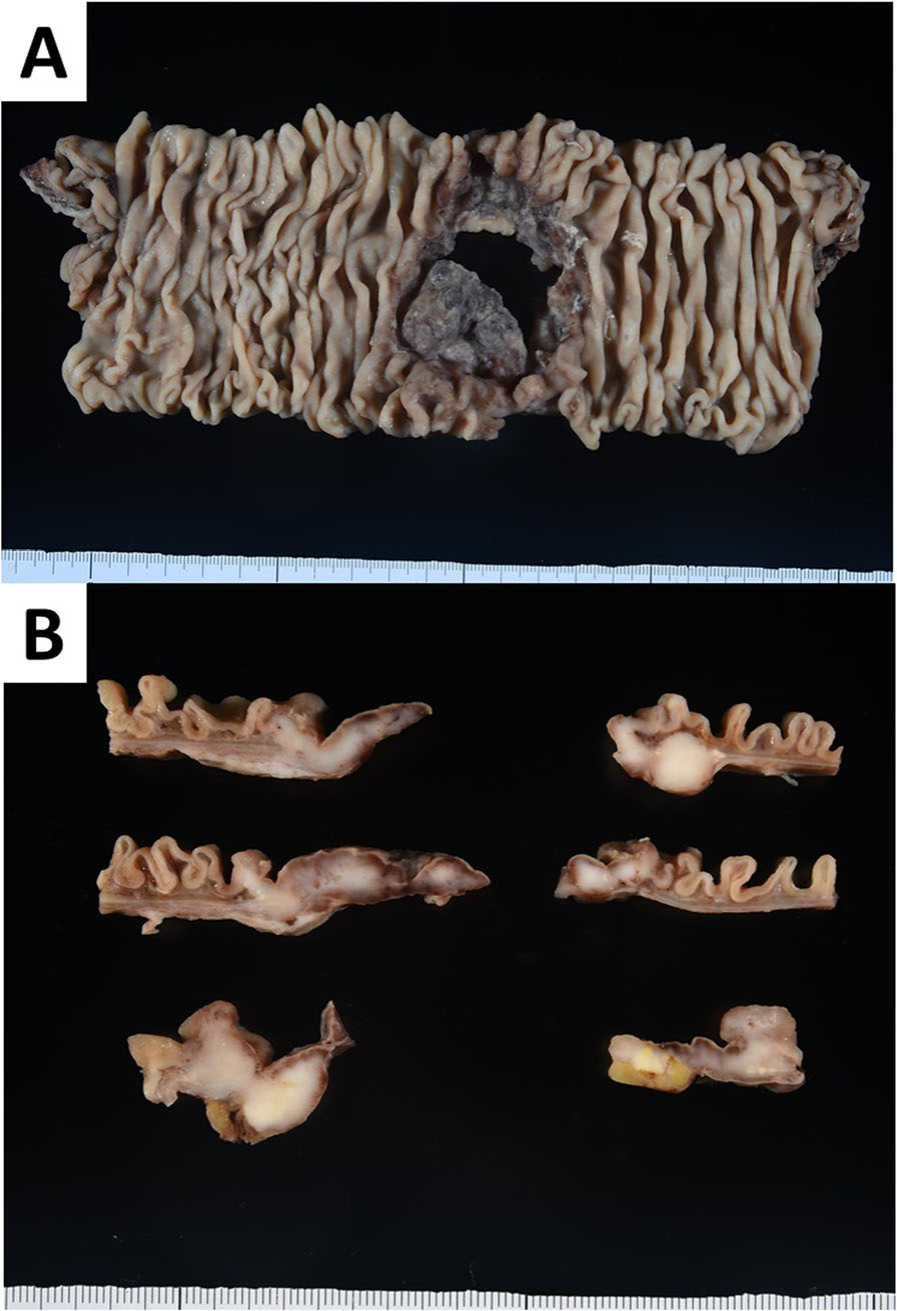
The resected small intestine of perforation site with lung adenocarcinoma metastasis. **a** There was about 5 × 4 cm perforation site with fibrotic change. **b** White tumors were found in the section of the small intestinal perforation site

**Fig. 3:**
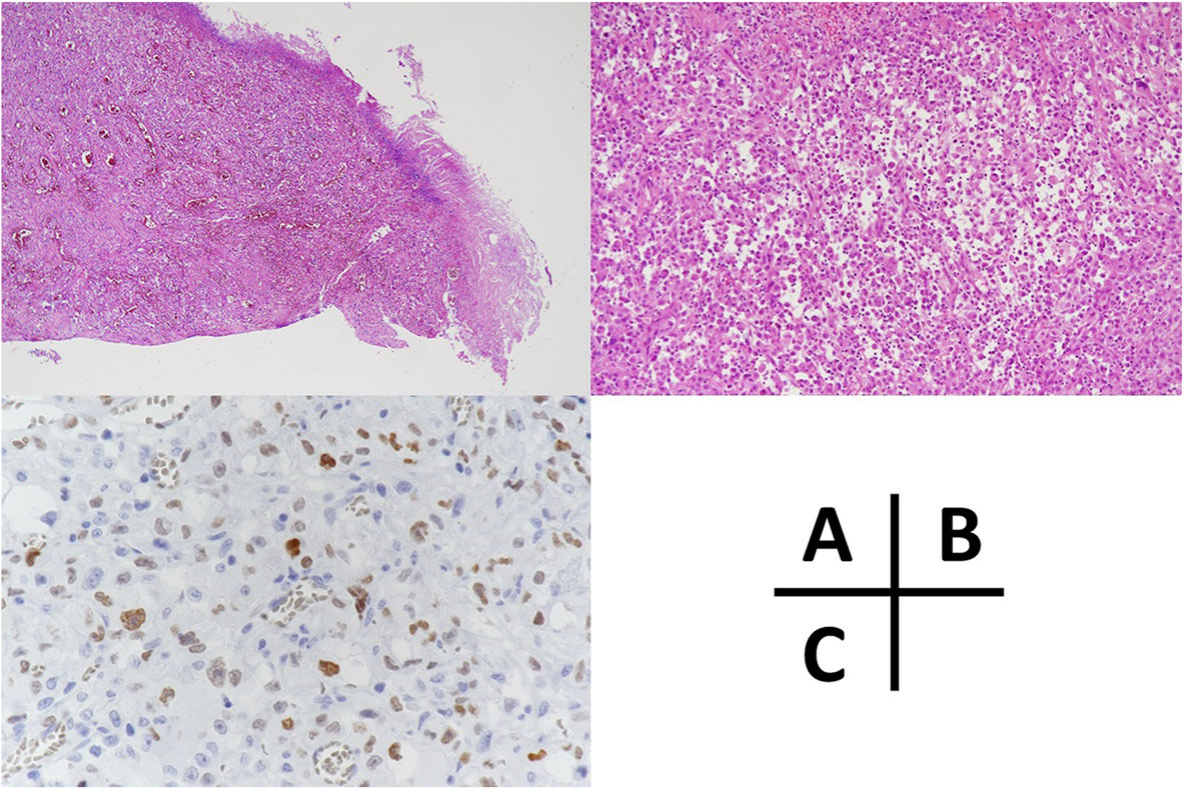
Pathological examination and immunohistochemical stain for thyroid transcription factor 1 (TTF-1). **a** The right side of the picture was the perforated site, and tumor cells and fibrosis were observed throughout the whole layer (× 40). **b** There were tumor cells with high degree of degeneration and necrosis change in the perforation site (× 100). The pathological response for the efficacy of pembrolizumab was grade 1b (*Japanese Classification of the Colorectal Carcinoma*, seventh edition). **c** The tumor cells showed TTF-1 expression in immunohistochemical stain

A 62-year-old man was treated with pembrolizumab for right lung adenocarcinoma, which showed high PD-L1 expression (80%), with multiple intestinal, lymph node, and bone metastases. The TNM classification for NSCLC was cT2N3M1c (OSS, LYM, PER, OTH), stage IVB (eighth edition). Tumor reduction was observed, but pembrolizumab was stopped after three courses owing to drug-induced pneumonitis. Dexamethasone was used for the treatment of pneumonitis. One month after drug withdrawal, the patient was transported to the emergency department of our hospital with the complaint of severe stomachache. On physical examination, he had a rigid abdomen and generalized tenderness. His blood pressure was in the normal range (110/82 mmHg), the heart rate was elevated but regular at 100 beats per minute, and the body temperature was elevated at 38.9 °C. The peripheral capillary oxygen saturation was 98% at room air. Laboratory evaluation showed a high inflammatory response with a white blood cell count of 18,200/mm^3^ and C-reactive protein level of 20.8 mg/dL. CT examination showed abdominal free air and ascites with perforation of the existing lung adenocarcinoma metastasis (Fig. 1). We diagnosed bowel perforation with acute diffuse peritonitis. Emergency laparotomy was performed, and multiple small intestinal metastasis with mesenteric lymph node metastasis and ascites containing intestinal fluid were observed. The perforation site was located in the metastatic jejunum about 40 cm on the anal side from Treitz’s ligament. We resected this part about 20 cm and anastomosed with functional end-to-end anastomosis. There was no complication after surgery, and he was discharged on post-operative day 15. Pathological examination indicated lung adenocarcinoma metastasis in the perforated intestine, and the metastasis was partly scarred owing to the effect of pembrolizumab (Fig. 2). Tumor cells in the perforation site had a high degree of degeneration and necrosis, and the pathological response for the efficacy of pembrolizumab was grade 1b (*Japanese Classification of the Colorectal Carcinoma*, seventh edition) (Fig. 3). In the perforated part, the tumor cells were observed in all layers, but in the vicinity on the serosa side. Inflammatory change due to enteritis was not found in this site. Pembrolizumab was re-administrated about 1 month after discharge. To prevent drug-induced pneumonitis, dexamethasone was used daily.

## Discussion

This is the first case report of intestinal perforation caused by the effect of pembrolizumab for NSCLC intestinal metastasis. One case of small intestinal perforation caused by metastasis of melanoma treated with pembrolizumab was reported in Italy [6]. In this case, multiple intestinal metastases were occurred, and some tumors had lined the entire intestinal wall in CT. Perforation cases of NSCLC intestinal metastasis treated with general chemotherapy were reported, and emergency surgery was done [7–9].

The interaction between PD-1 receptor with its ligands, PD-L1 and PD-L2, regulates the balance between T cell activation, immune tolerance, and immune-related tissue damage, and in tumor environment, this pathway is taken over to evade immune surveillance [10, 11]. Pembrolizumab is a highly selective humanized immunoglobulin G4/κ monoclonal antibody designed to directly inhibit between PD-1 receptor and PD-L1/PD-L2 interaction by binding to PD-1 receptor. In KEYNOTE-024 trial, advanced NSCLC patient group treated with pembrolizumab had longer progression-free survival than those treated with chemotherapy, and the median progression-free survival was 10.3 months vs. 6.0 months, respectively (*P* < 0.001) [4].

Anti-PD-1 antibodies, including pembrolizumab, have many side effects ranging from leukopenia, nausea, and fatigue, which are commonly seen with other anti-cancer drugs, to side effects attributed to the autoimmune response to this drug like hypothyroidism, type 1 diabetes, hypopituitarism, colitis, and drug-induced pneumonitis [4, 5]. The patient in our case also experienced drug-induced pneumonitis, which was treated with dexamethasone. Hence, there was an increased risk of perioperative infection (surgical site infection and abdominal abscess) and anastomosis failure. Fortunately, there were no major complications or side effects due to excessive immune reaction. Generally, characteristic side effects of pembrolizumab occur immediately after administration. However, it is not uncommon to see the side effects after a few months [5]. Patients who have been treated with pembrolizumab should always consider autoimmune side effects, and it is necessary to treat these as soon as their onset is suspected.

## Conclusions

This is the first report of perforation of NSCLC small intestinal metastasis after pembrolizumab treatment. It is necessary to perform perioperative management with the onset of autoimmune side effects characteristic of anti-PD-1 antibodies, including pembrolizumab.

## Data Availability

The datasets supporting the conclusions of this article are included within this article.

## Abbreviation

CT: Computer tomography
